# A systematic review of the case findings, testing and management of COVID-19

**DOI:** 10.12688/f1000research.50929.2

**Published:** 2022-02-02

**Authors:** Dewi Susanna, Dian Pratiwi, Sang Gede Purnama

**Affiliations:** 1 Department of Environmental Health, Faculty of Public Health, Universitas Indonesia, Depok, Jawa Barat, 16424, Indonesia; 2 Alumni of Faculty of Public Health, Universitas Indonesia, Depok, Jawa Barat, 16424, Indonesia; 3 Faculty of Medicine, Udayana University, Denpasar, Bali, 80234., Indonesia; 4 Doctoral Program in Faculty of Public Health, Universitas Indonesia, Depok, Jawa Barat, 16424, Indonesia

**Keywords:** COVID-19, SARS-CoV-2, case detection mechanism, case finding, case management, diagnostic test

## Abstract

**Background:** Mass testing and adequate management are essential to terminate the spread of coronavirus disease 2019 (COVID-19). This testing is due to the possibility of unidentified cases, especially ones without COVID-19 related symptoms. This review aimed to examine the outcome of the existing studies on the ways of identifying COVID-19 cases, and determine the populations at risk, symptom and diagnostic test management of  COVID-19.

**Methods:** The articles reviewed were scientific publications on the PubMed, Science Direct, ProQuest, and Scopus databases. The keywords used to obtain the data were COVID-19, severe acute respiratory syndrome coronavirus 2 (SARS-CoV-2) and case detection, case management or diagnostic test. We applied the Preferred Reporting Items for Systematic Reviews and Meta-Analyses (PRISMA) and Population, Intervention, Control and Outcomes (PICO) approaches.

**Results:** A total of 21 articles from 13 countries met the inclusion criteria and were further analyzed qualitatively. However, 62% of the articles used a rapid antibody test for screening rather than a rapid antigen test. According to the rapid antigen test, 51.3% were positive, with men aged above 50 years recording the highest number of cases. Furthermore, 57.1% of patients were symptomatic, while diagnostic tests' sensitivity and specificity increased to 100% in 14 days after the onset.

**Conclusion** **s**:  Real-time polymerase chain reaction (RT-PCR)  is recommended by the World Health Organization for detection of COVID-19. Suppose it is unavailable, the rapid antigen test is used as an alternative rather than the rapid antibody test. Diagnosis is expected to be confirmed using the PCR and serological assay to achieve an early diagnosis of COVID-19, according to disease progression, gradual rapid tests can be used, such as rapid antigen in an earlier week and antibody tests confirmed by RT–PCR and serological assay in the second week of COVID-19.

## Introduction

The outbreak of severe acute respiratory syndrome coronavirus 2 (SARS-CoV-2) is known as coronavirus disease 2019 (COVID-19). This outbreak started in Wuhan Hubei, China, in early December 2019 ^1^. Presently, an exponential increase in infection cases has been continuously reported in various countries, although vaccinations now accompany this.

Coronaviruses are a group of RNA viruses that cause various respiratory, gastrointestinal, and neurological diseases with mild and severe symptoms in humans and animals. There are at least two types with severe symptoms: Middle East respiratory syndrome (MERS) and severe acute respiratory syndrome (SARS). COVID-19, which SARS-CoV-2 causes, is a new type of disease that humans have never identified before. Furthermore, it is regarded as a zoonotic disease (an animal disease transmitted to humans). SARS has been reported in several studies to be transferred from civets to humans while MERS is contacted from camels. Meanwhile, the particular animal source of COVID-19 transmission is still unknown ^2^.

The governments of various countries have created several services to handle and prevent the spread of COVID-19. Furthermore, several steps have been taken, such as the rapid purchase of test kits, additional health facilities to accommodate patients, laboratories capable of examining blood specimens, human resources, equipment, infrastructure, etc. It is presumed that these additions can suppress the number of positive cases. Patients with symptoms are immediately tested and treated or even monitored; however, the number of positive cases is still increasing. 

The strategies for the prevention and control of COVID-19 include increasing epidemic surveillance, quarantining the infection source, speeding up the diagnosis of suspected cases, optimizing close contact management, constricting the prevention and control of outbreaks. The strategies also prevent possible epidemic rebounds by immediate quarantine of individuals in close contact of positive cases and strengthening community prevention and control measures ^3^. However, early and accurate case findings are necessary to maximize these efforts. Therefore, it is important to review the results of existing studies on finding cases, determine the population at risk, determine diagnostic tests, and provide facilities including human resources and tools to prevent Covid-19 transmission to ending this pandemic.

Due to the influence of COVID-19, several studies have recently been conducted because the pandemic is complex in many aspects of life ^4– 6^. The complexity is attributed to the crises experienced in the national health, economic, education, cultural, sports, and social systems ^6^, apart from the drug and vaccine candidates ^5^. The occurrence and development of SARS-CoV-2 depend on the interaction between the virus and the individuals' immune system ^7^. Therefore, its treatment requires special analyses for case findings and management of COVID-19 cases. Presently, there is a controversy over the use of rapid tests and screening for new cases. For instance, Indonesia's government is yet to decide whether rapid tests need to be continued or stopped.

This review aimed to examine the variations in COVID-19 diagnostic testing and clinical characteristics across various studies.

## Methods

A systematic review was conducted to identify articles that describe the diagnostic, identification, and management of SARS-CoV-2 and COVID-19 cases. The review is reported following the Preferred Reporting Items for Systematic Reviews and Meta-Analyses (PRISMA) guidelines ^8^.

### Ethical approval

This review received ethical approval from the Research and Community Engagement Ethical Committee of Faculty of Public Health, Universitas Indonesia Number: Ket-198/UN2.F10.D11/PPM.00.02/2020.

### Inclusion criteria and exclusion criteria

Original studies published in open-access journals before 1 August 2020 in English during the COVID-19 pandemic were included. Studies had to include rapid COVID-19 tests and screening. Closed access articles, audio, communication, reviews, reports, perspectives, case studies, surveys, clinical and molecular papers, mathematical modelling, and diagnostic procedures were excluded from this review.

### Search strategy

A systematic search was conducted in four databases, specifically Scopus, Science Direct, ProQuest and PubMed. The keywords used to obtain data from Science Direct included COVID-19/COVID/coronavirus 2019 OR SARS-CoV-2 AND rapid test OR rapid diagnostic test AND screening.

For Scopus, the following search was used: ((TITLE-ABS-KEY (covid-19) OR TITLE-ABS-KEY (covid) OR TITLE-ABS-KEY (coronavirus 2019) OR TITLE-ABS-KEY (sars-cov-2) AND TITLE-ABS-KEY (rapid AND test) OR TITLE-ABS-KEY (covid AND diagnostic AND test) AND TITLE-ABS-KEY (screening)).

The search used for Science Direct was "COVID-19" OR COVID OR "coronavirus 2019" OR "SARS-CoV-2" AND ("rapid test" OR "rapid diagnostic test") screening. The ProQuest used covid-19 OR covid OR (coronavirus 2019) OR (sars-cov-2) AND (rapid test) OR (rapid diagnostic test) AND (screening).

The search used for PubMed was ((((covid-19) OR (covid) OR (coronavirus 2019) OR (SARS-CoV-2)) AND (rapid test)) OR (rapid diagnostic test)) AND (screening)))).

### Study selection

The initial screening was conducted for articles between 1 December 2019 and 31 July 2020. All the authors recorded and reviewed the collected articles. Furthermore, DS determined the study design, time frame, and criteria for the included studies to retrieve the articles and process the data. Importantly, DS retrieved the data from Scopus and Science Direct databases, while DP from ProQuest and PubMed databases as SGP's suggestion. The identified articles' information was imported into Excel worksheets and Mendeley Desktop, where duplicates between databases were removed. DS, DP, and SGP separately reviewed the titles to eliminate analysis that does not meet the inclusion criteria before reviewing the title and the abstract. Moreover, DS and DP accessed the full-text articles for the eligibility criteria. In case there were differences in the number of articles obtained, the two authors re-checked the articles again using the same criteria until the same articles are selected. The final decision of articles included was after DS and DP. All authors discussed the variables to assess the full paper using PICOS to determine the study questions. The PICOS's assessment used include 1) Population, 2) Intervention: The diagnostic tests for COVID-19, 3) Comparison: the method of the test and antigen or antibody results, and 4) Outcome: The antibody or antigen tests. Any discrepancies were resolved through consensus via a virtual meeting.

Quality assessment for the selected articles was performed using a modified checklist ^9^ that consisted of seven questions. If the answer of the question is ‘yes’, the value will be ‘1’, while if the answer is ‘no’, the value will be ‘0’. Each article will have a total value, then scored (in %) by calculating the total value divided by the total number of question, then multiplied by 100.

The score grouped into three scoring (in %) = total score divided by the total number of question, then multiplied by 100; then categorized as ‘good’ (68–100%), satisfactory (34–67%), and bad (0–30%), as shown in Table 1 (as an attachment).

**Table 1.** Quality assessment of the selected articles ^9^.

	**1**	**2**	**3**	**4**	**5**	**6**	**7**	**8**	**9**	**10**	**11**	**12**	**13**	**14**	**15**	**16**	**17**	**18**	**19**	**20**	**21**
The value of each yes = 1																					
The value of each no = 0																					
Questions:																					
1. Are the aims/ objectives clearly described?	1	1	1	1	1	1	1	1	1	1	1	1	1	1	1	1	1	1	1	1	1
2. Is the sampling process described?	1	1	1	1	1	1	1	1	1	1	1	1	1	1	1	1	1	1	1	1	1
3. Is the research methods described?	1	1	1	1	1	1	1	1	1	1	1	1	1	1	1	1	1	1	1	1	1
4. Is the data collection process described?	1	1	1	1	1	1	1	1	1	1	1	1	1	1	1	1	1	1	1	1	1
5. Are the findings described and explained?	1	1	1	1	1	1	1	1	1	1	1	1	1	1	1	1	1	1	1	1	1
6. Is the symptom of COVID-19 compared?	0	0	0	0	1	0	0	1	1	1	1	1	0	1	1	1	1	0	0	1	1
7. Is the diagnostic test described? (immunology assay compared)	0	0	1	1	0	1	1	1	0	0	1	1	0	1	1	0	0	1	1	0	0
**Total**	5	5	6	6	6	6	6	7	6	6	7	7	5	7	7	6	6	6	6	6	6
**%**	71.4	71.4	85.7	85.7	85.7	85.7	85.7	100.0	85.7	85.7	100.0	100.0	71.4	100.0	100.0	85.7	85.7	85.7	85.7	85.7	85.7
**Average**	87.1																				

[i] *Scoring (in %) = total score divided by the total number of question, then multiplied by 100; then categorized as good (68–100%), satisfactory (34–67%), and bad (0–30%)*

### Data extraction

All authors designed the variables to be described in the matrix and the topics discussed. The differences in the case detection method of the articles could be a source of bias. To minimize the biases, this review determined and selected the same variables (screening, symptoms, and diagnostic tests; at least an article had one of the following epidemiological parameters regarding COVID-19 or SARS-CoV-2: (i) signs and symptoms, (ii) types of test, (iii) case findings, (iv) screening and testing for COVID-19, (v) procedures for managing positive cases, or (vi) interventions or treatments.). The diagnostic test of each article was different after-time onset. Therefore, the authors used the limit after onset both less and more than 14 days (after onset ≤ 14 days and > 14 days).

### Data analysis

The outcome of the analysis was displayed in a matrix containing the author's name, title, date of research, country of origin of the article, method, and results. Another table included the symptoms, incubation period, method of case finding, diagnostic tests, and type of examination in a more detailed manner. Management identification was based on the type of intervention, care, and treatment.

Descriptive synthesis conducted in the textual description of findings and presented in all tables. A narrative synthesis was undertaken for analysis based on the topics selected. The issues raised included high-risk group, case findings, symptoms of COVID-19 patients, diagnostic test, and the potential strengths and weaknesses of this review.

## Results

**Figure 1.**
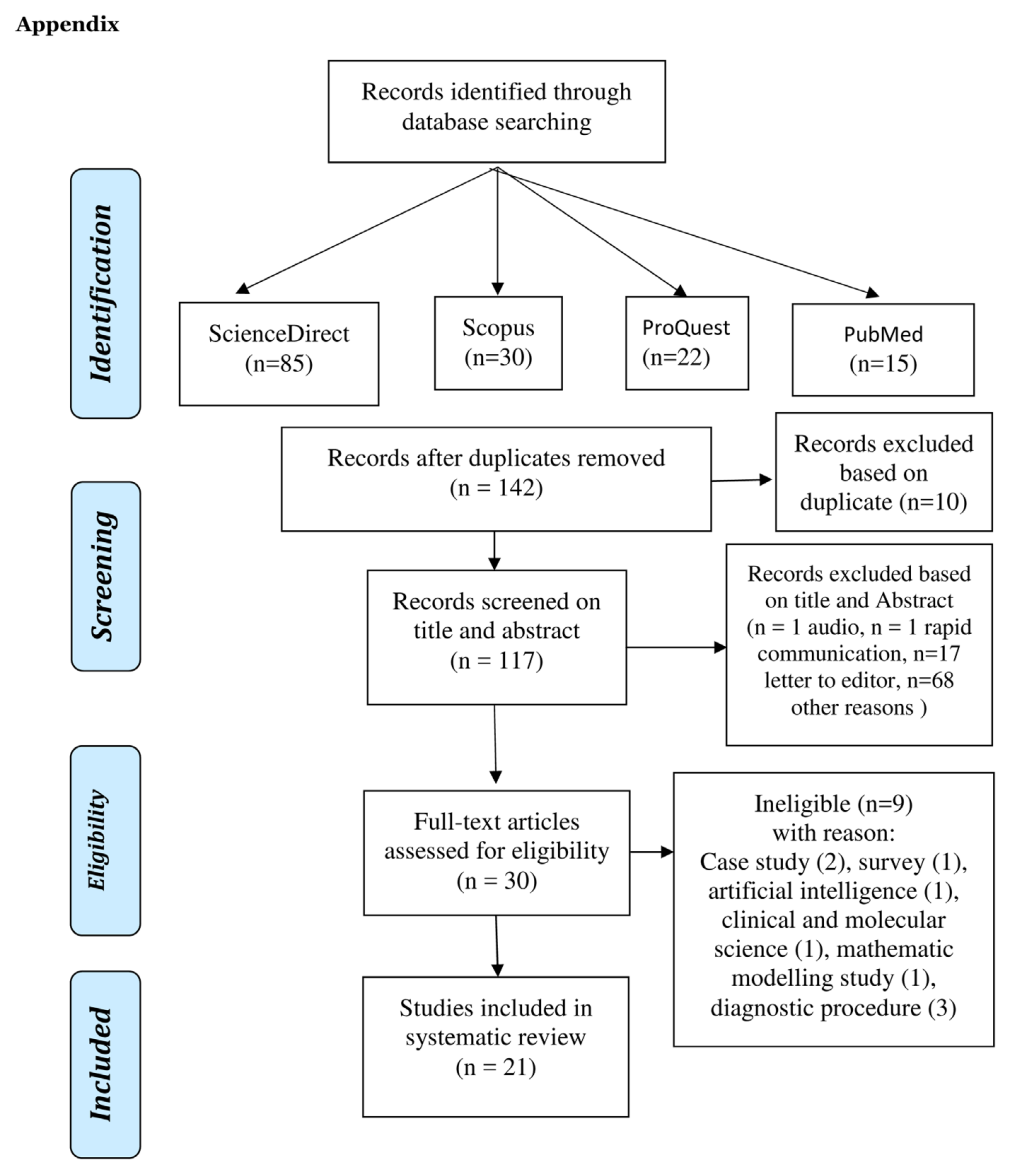
Flow chart of the search strategy and article selection.

Based on the four databases, 152 titles were obtained, with 21 studies included in the review. Figure 1 provides an overview of the articles included.

The assessment of articles selected showed that the average quality score was 87.1% and ranged from 71.4% to 100% ( Table 1).

There were 21 eligible articles conducted between December 2019 and 31 July 2020 from 13 countries, including France, the United States of America, Italy, Singapore, Chile, Germany, Taiwan, South Korea, Austria, Bulgaria, Japan, Spanish, and Brazil ( Table 2).

**Table 2.** Clinical and demographic characteristics of studies included in the systematic review.

Author	Title	Research Time	Country	Methods - design	Number of samples
Kimbal A *et al.*	Asymptomatic and Presymptomatic SARS-CoV-2 Infections in Residents of a Long-Term Care Skilled Nursing Facility King County, Washington, March 2020 ^14^	28 Feb-27 March 2020	United States of America	SARS-CoV-2 test	76
Lorenzo and Carrisi	COVID-19 exposure risk for family members of healthcare workers: An observational study ^15^	2-31 May 2020	Italy	Observational study	99
Tuaillon *et al.*	Detection of SARS-CoV-2 antibodies using commercial assays and seroconversion patterns in hospitalized patients ^16^	18 March 2020	France	SARS-CoV-2 antibodies using commercial assays	58
Demey *et al.*	Dynamic proﬁle for the detection of anti-SARS-CoV-2 antibodies using four immunochromatographic assays ^17^	2020	France	Immunochromatographic assays	22
Sun *et al.*	Epidemiological and Clinical Predictors of COVID-19 ^18^	26 Jan-16 Feb 2020	Singapore	Epidemiological and Clinical test	788
Margiotti *et al.*	Evaluation of A Rapid IgM-IgG Combined Antibody Test for SARS- CoV-2 Infection: Single Italian Center Study ^19^	2020	Italy	Antibody Test	194
Porte *et al.*	Evaluation of a novel antigen-based rapid detection test for the diagnosis of SARS-CoV-2 in respiratory samples ^20^	2020	Chile	Antigen-based rapid detection test	127
Wu *et al.*	Four point-of-care lateral ﬂow immunoassays for diagnosis of COVID-19 and for assessing dynamics of antibody responses to SARS-CoV-2 ^21^	23 Jan-25 April 2020	Taiwan	Rapid lateral flow immunoassay	46
Cho *et al.*	Hemodialysis with Cohort Isolation to Prevent Secondary Transmission during a COVID-19 Outbreak in Korea ^22^	20 Jan-14 March 2020	Korea	Cohort study	302
Nepogodiev D *et al.*	Mortality and pulmonary complications in patients undergoing surgery with perioperative SARS-CoV-2 infection: an international cohort study ^23^	1 Jan- 31 March 2020	24 countries	Cohort study	1128
Weidner *et al.*	Quantiﬁcation of SARS-CoV-2 antibodies with eight commercially available immunoassays ^24^	2020	Austria	SARS-CoV-2 antibodies immunoassay	100
Döhla M *et al.*	Rapid point-of-care testing for SARS-CoV-2 in a community screening setting shows low sensitivity ^25^	2020	Germany	Rapid point-of-care testing for SARS-CoV-2	49
Azzi *et al.*	Saliva is a reliable tool to detect SARS-CoV-2 ^26^	2020	Italy	SARS-CoV-2 test using saliva	25
Tsaneva- damyanova	SARS-CoV-2: seroepidemiological pattern in northeastern Bulgaria ^27^	26 March-20 April 2020	Bulgaria	Seroepidemiological	586
Banerjee *et al.*	Use of Machine Learning and Artificial Intelligence to predict SARS-CoV-2 infection from Full Blood Counts in a population ^28^	28 March-30 April 2020	Brazil	Machine Learning and Artificial Intelligence	598
Liu *et al.*	Antibody responses against SARS-CoV-2 in COVID-19 patients ^29^	January 26 and 8 March, 2020	China	Retrospective study	42
Zhou *et al.*	The dynamic changes of serum IgM and IgG against SARS-CoV-2 in patients with COVID-19 ^30^	January 26 to 5 March, 2020	China	Retrospective study	97
Kaneko *et al.*	Clinical validation of an immunochromatographic SARS-Cov-2 IgM/IgG antibody assay with Japanese cohort ^31^	March and May 2020	Japan	Cohort study	51
Yu *et al.*	Distinct features of SARS-CoV-2-specific IgA response in COVID-19 patients ^32^	2020	China	Immunoassay	37
de la Iglesia *et al.*	Concordance between two rapid diagnostic tests for the detection of antibodies against SARS-CoV-2 ^33^	2020	Spanish	Cross-sectional study	110
Sotgiu *et al.*	SARS-CoV-2 specific serological pattern in healthcare workers of an Italian COVID-19 forefront hospital ^34^	April 2 to 16 April, 2020	Italy	Immunoassay	202

[i] *COVID-19:coronavirus disease 2019 ELISA : Enzyme linked immunosorbent assay IgA : Immunoglobulin A IgG : Immunoglobulin G IgM : Immunoglobulin M SARS-CoV-2: severe acute respiratory syndrome coronavirus 2*

Each country has its reasons and policies for adopting the coronavirus screening method. Besides polymerase chain reaction (PCR), antigen and antibody tests, there are various tests for detecting the virus, such as the use of clinical immunoassays. An immunoassay is a biomedical test for measuring molecules' presence in a solution through the use of antibodies or antigens ^10^.

A rapid test is the screening method for detecting COVID-19 that shows the results quickly, specifically between a few minutes to a maximum of one hour. The methods in Table 3 are divided into two, namely the antigen and antibody rapid tests. The rapid antigen test is used to detect a viral protein (antigen) and is detected when the virus is actively replicating. Conversely, the rapid antibody test is used to detect antibodies or immunoglobins produced by the body against the virus. According to Table 3, showing the screening tests used in the studies, 62% of the articles (13/21) adopted the rapid antibody and antigen tests, for mostly males aged over 50 years. The rapid antigen test results showed that 51.3% were positive, as shown in Table 3.

**Table 3.** Screening tests used to detect COVID-19.

No	Author	Rapid antigen test (swab)			Rapid antibody test (blood- serum/plasma)			Average Age (years)	Gender (%)	
		n	Positive (n)	Positive (%)	n	Positive (n)	Positive (%)		Male	Female
1	Kimball A *et al.*	76	23	30.3	-	-	-	80.7	0	100
2	Sun *et al.*	788	54	6.9	-	-	-	42	53.7	46.3
3	Porte *et al.*	127	82	64.6	-	-	-	38	53.7	46.3
4	Cho *et al.*	302	18	6.0	-	-	-	55.5	44.4	55.6
5	Nepogodiev D *et al.*	1128	1128	100.0	-	-	-	70	52.8	47.2
6	Azzi *et al.*	25	25	100.0	-	-	-	61.5	68	32
7	Liu *et al.*	42	42	100.0	-	-	-	61	33.3	66.7
8	Sotgiu *et al.*	202	7	3.5	-	-	-	45	34.7	65.3
9	Lorenzo and Carrisi	-	-	-	99	28	28.3	47	NA	NA
10	Tuaillon *et al.*	-	-	-	58	38	65.5	65–72	57.8	42.2
11	Demey *et al.*	-	-	-	22	22	100.0	NA	NA	NA
12	Margiotti *et al.*	-	-	-	194	132	68.0	35.5	42.4	57.6
13	Wu *et al.*	-	-	-	46	16	34.8	45.6	56.3	43.7
14	Weidner *et al.*	-	-	-	100	98	98.0	47	61	39
15	Döhla M *et al.*	-	-	-	49	22	44.9	46	51	49
16	Tsaneva-damyanova	-	-	-	586	28	4.8	45	35.7	64.3
17	Banerjee *et al.*	-	-	-	598	81	13.5	NA	NA	NA
18	Zhou *et al.*	-	-	-	97	97	100.0	65	NA	NA
19	Kaneko *et al.*	-	-	-	51	51	100.0	63	72.5	27.5
20	Yu *et al.*	-	-	-	37	37	100.0	52	67.6	32.4
21	de la Iglesia *et al.*	-	-	-	110	58	52.7	48	48	52
Total		2690	1379	51.3	2047	708	34.6			

[i] *Legend: NA= Not Available*

The terms for COVID-19 patients are divided into several groups, namely patients under monitoring or confirmed cases without symptoms ^11^. The clinical manifestations of COVID-19 patients have a broad spectrum, ranging from lack of symptoms to mild illnesses, pneumonia, severe pneumonia, and septic shock. The characteristics of the symptoms shown are in accordance with the results of the journals reviewed. In Table 4, the symptoms were divided into two groups, namely typical and atypical. Typical symptoms are the most frequently reported clinical manifestations. The virus enters through the nose and mouth and attacks the respiratory tract with typical symptoms, namely fever > 38°C and cough ^12^. Conversely, atypical symptoms are clinical manifestations originating from organs other than the lungs. The reactive results from patients examined by the rapid test showed that 42.9% were asymptomatic or lacked available data. In comparison, 57.1% were symptomatic, with typical symptoms such as fever, cough, respiratory syndrome, sore throat, pneumonia, loss of taste and smell, and atypical symptoms including malaise, nausea, gastrointestinal disorders, headaches, and fatigue, as shown in Table 4.

**Table 4.** Symptoms of the COVID-19 patients.

Author	Positive COVID-19 (n)	Symptomatic										
		Proportion of patients with typical symptoms (%)						Proportion of patients with atypical symptoms (%)				
		Fever	Cough	Respiratory tract syndrome	Loss of taste and smell	Sore throat	Pneumonia	Malaise	Nausea	Gastrointestinal disease	Headache myalgia	Fatigue
Kimball A *et al.*	23	NA	NA	NA	NA	NA	NA	NA	NA	NA	NA	NA
Lorenzo and Carrisi	28	NA	NA	NA	NA	NA	NA	NA	NA	NA	NA	NA
Tuaillon *et al.*	38	NA	NA	NA	NA	NA	NA	NA	NA	NA	NA	NA
Demey *et al.*	22	NA	NA	NA	NA	NA	NA	NA	NA	NA	NA	NA
Sun *et al.*	54	37.5	66.7	13	NA	NA	42.6	NA	NA	37	NA	NA
Margiotti *et. al*	132	NA	NA	NA	NA	NA	NA	NA	NA	NA	NA	NA
Porte *et al.*	82	NA	NA	NA	NA	NA	NA	NA	NA	NA	NA	NA
Wu *et al.*	16	50	NA	75	NA	NA	62.5	NA	NA	18.8	31.3	NA
Cho *et al.*	18	55.6	NA	NA	NA	NA	NA	NA	NA	NA	NA	NA
Nepogodiev D *et al*.	1128	76.6	73	61.9	NA	NA	NA	NA	79.4	77.5	79.4	70
Weidner *et al.*	98	63	40	NA	43	29	NA	NA	NA	23	48	NA
Döhla M *et al.*	22	NA	70.8	NA	NA	NA	NA	NA	NA	NA	NA	64.6
Azzi *et al.*	25	NA	NA	NA	NA	NA	NA	NA	NA	NA	NA	NA
Tsaneva-damyanova	28	NA	66.7	NA	NA	NA	NA	NA	NA	NA	NA	NA
Banerjee *et al.*	81	40	40	NA	NA	NA	40	NA	NA	NA	NA	NA
Liu *et al.*	42	66.7	52.4	21.4	NA	11.9	NA	NA	NA	NA	NA	NA
Zhou *et al.*	97	59.8	86,6	NA	NA	NA	NA	NA	NA	NA	NA	71,1
Kaneko *et al.*	51	NA	NA	NA	NA	NA	NA	NA	NA	NA	NA	NA
Yu *et al.*	37	NA	NA	NA	NA	NA	NA	NA	NA	NA	NA	NA
de la Iglesia *et al.*	58	35.5	NA	NA	41.8	NA	NA	NA	NA	NA	NA	NA
Sotgiu *et al.*	7	4.5	4.5	NA	1.5	4	NA	NA	NA	NA	NA	NA

[i] *Legend: NA= Not Available*

The rapid test is verified through a diagnostic test to confirm the patients' status, whether positive or negative. Real-time polymerase chain reaction (RT-PCR) testing of SARS-CoV-2 has become a standard method for direct diagnosis. Currently, RT-PCR is used to diagnose COVID-19 by detecting genetic material of the coronavirus ^13^. Serologic and immunological tests such as ELISA (enzyme linked immunosorbent assay), POC or LFA (point-of-care lateral flow assay), and CLIA (chemiluminescence immunoassay) complement RT-PCR examinations in screening and diagnosis of COVID-19.

Meanwhile, the POC or LFA is a type of rapid examination for diagnosing infectious diseases and the results are shown within minutes, permitting quick decisions regarding the patients' care. POC also extends its testing to communities and populations that do not have easy access to health care ^35^. ELISA is an analytical biochemical test that is used to evaluate the presence of an antigen or antibody in a sample. It is useful in the determination of serum antibody concentration ^36^. CLIA is the assay for detection antibodies against the SARS-CoV-2 nucleoprotein (Np) in serum or plasma.

Only 11 articles out of 21 titles provided sensitivity or specificity data ( Table 5a and Table 5b). At 14 days after symptom onset, the test results were in IgG, IgM, and IgA (antibody) values, because at that particular time-point, antibodies have formed. Immunoglobin M (IgM) tends to increase within 3–14 days after infection and is replaced by Immunoglobin G (IgG) for 7 to 15 days, which tends to remain detectable for months. Meanwhile, immunoglobin A (IgA) is usually used to diagnose disorders in the immune system and detect mucosal secretions such as saliva. The sensitivity indicates the ability of the test to show a positive result. Therefore, the higher the test sensitivity, the greater the positive test results, and the lesser the number of false negatives.

**Table 5a.** Sensitivity and specificity of diagnostic tests used in the reviewed articles.

Author	≤14 days after onset (%)				>14 days after onset (%)			
	RT PCR	ELISA	POC LFA	CLIA	RT PCR	ELISA	POC LFA	CLIA
Porte *et al.*	se: 80-94,7							
	sp:100							
Tuaillon *et al.*		se:43-86(IgA)	se:36-93(IgG)			se:80-100(IgA)	se:80-100(IgG)	
		se:36-93 (IgG)	se:36-86 (IgM)			sp:80 (IgA)	sp:95-100 (IgG)	
						se:73-100 (IgG)	se:73-100 (IgM)	
						sp:85-100 (IgG)	sp:65-100(IgM)	
Kaneko *et al.*		se:81,6 (IgG)						
		se:71 (IgM)						
Demey *et al.*			se:9,09-100 (IgG)				se:81,82-100 (IgG)	
			se:4,55-100 (IgM)				se:100 (IgM)	
Wu *et al*			se:41,3-52,2 (NA)				se:87-100(NA)	
			sp:100 (NA)				sp:100 (NA)	
Yu *et al.*				se: 98,9 (IgA)				se: 100 (IgA)
				se:95,1 (IgG)				se:100 (IgG)
				se:93,4(IgM)				se:100 (IgM)

Specificity indicates a test's ability to show a negative result for individuals who do not have the virus. Therefore, the higher it is, the more negative test results, or the fewer false positives ^37^. Overall, the sensitivity and specificity tend to be accurate or have high values after 14 days of onset with 100 for all immunoassay assays, as shown in Table 5a and Table 5b.

**Table 5b.** Sensitivity and specificity of diagnostic tests used in the reviewed articles.

Author	Not Available days after onset			
	RT PCR	ELISA	POC LFA	CLIA
Margioti *et al.*	se:95,5(NA)			
	sp:96,8(NA)			
Döhla M *et al.*	se:36,4(NA)			
	sp:88,9(NA)			
Banerjee *et al.*	se:43-92(NA)			
	sp:58-94(NA)			
Weidner *et al.*		se:88,89-98(NA)	se:88.78-92,93(NA)	se:84,94-95(NA)
Tsaneva damyanova			se:100(IgG)	
			sp:98(IgG)	
			se:85(IgM)	
			sp:96(IgM)	

[i] *Se: sensitivity Sp: specificity RT-PCR : Real time-polymerase chain reaction ELISA: Enzyme-linked immunosorbent assay POC LFA: Point-of-care lateral flow assay CLIA: Chemiluminescence immunoassay NA=Not available*

## Discussion

### Comorbidities

Indonesia's government implemented a rapid test policy to accelerate the early detection of confirmed cases, both among health workers and other high-risk groups. However, this test has drawbacks because positive results are only obtainable among individuals with COVID-19 antibodies in their blood, which are generally formed on the seventh day after infection. Consequently, there is a possibility of the result being negative but does not mean that the individual is not infected. This occurrence is since the antibodies are yet to be formed; therefore, repetition is needed. The implementation of the rapid test is intended for individuals that are at risk. However, in this current condition, mass testing could be carried out considering the number of infected people without symptoms that have not received treatment and monitoring, which are all sources of transmission.

The elderly and individuals with pre-existing medical conditions such as high blood pressure, heart and lung disorders, diabetes, and cancer are at greater risk of experiencing severe COVID-19 symptoms ^38^. Furthermore, travelers and individuals who have had close contact with infected individuals and medical personnel ^39^. Therefore, surveillance for this group needs to be carried out daily with active case finding through screening for signs and symptoms and checking body temperature ^5^. Based on gender distribution, males are presumed to be associated with a higher prevalence of active smoking ^39^. It is suspected that there is an increase in ACE2 receptor expression in smokers, people with hypertension, and diabetes mellitus ^39, 40^.

COVID-19 patients with other comorbidities such as chronic obstructive pulmonary disease (COPD), cardiovascular disease (CVD), hypertension, cancer, diabetes, HIV, chronic kidney disease can cause a high risk of death. Comorbidities cause COVID-19 patients to be more at risk of increasing morbidity and mortality ^41– 43^. A cohort study in Jakarta also found a higher risk of death with comorbid patients than those without, the risk increasing sixfold among patients <50 years of age ^44^. Therefore, comorbidities can exacerbate COVID-19 infection ^45^.

### Case findings

The COVID-19 pandemic has been driven by cross-border human mobility and region-specific COVID-19 susceptibilityy ^46^. The diagnosis of new cases is inseparable from early precautions ^2^. One method of how a diagnosis is carried out is via screening. During the COVID-19 pandemic, screening at airports has been a priority due to its spread in 113 countries globally, which allegedly started in Wuhan (China). Initially, it was only a thermal test developed into a quarantine system at airports or ports. While active screening at airports is still an effective method for detecting new diseases, it does not provide 100% efficacy in case detection ^47^ because there are passive cases that are yet reported at health services.

Surveillance activities may be either passive or active. In passive surveillance, the health department passively receives reports of suspected injury or illness. Conversely, epidemiologists actively seek out cases of disease ^48^. The detection of passive cases is triggered by patients seeking to be treated by doctors working in health facilities. Meanwhile, active screening detects 80% and 20% of imported and passive cases, respectively ^47^.

The active case findings under rapid tests in the community, for instance, in Indonesia, are currently being carried out by inviting individuals to various designated places, such as the health office, stadium, village centers, markets, and schools. South Korea adopted a test kit from SD Biosensor to carry out mass testing in its country as a preventive. This test has proven to be a practical rapid screening step, consequently reducing the death rate. However, this rapid test is also supported by the PCR test with free drive-through service. The test kit's performance is influenced by several factors, such as the period of emergence of symptoms, the concentration of virus in the specimen, quality and method of processing, and the reagent formulation in test kit ^49^.

### Symptoms of COVID-19 patients

The terms for COVID-19 patients are divided into several groups, namely patients under monitoring (ODP) or close contacts, patients under supervision (PDP) or suspected cases, and patients without symptoms (OTG) or confirmed cases without symptoms ^11^. The clinical manifestations of COVID-19 have a broad spectrum, ranging from asymptomatic, mild symptoms, pneumonia, severe pneumonia, acute respiratory distress syndrome (ARDS), sepsis to septic shock. Approximately 80% of cases have been classified as mild or moderate, 13.8% as severe, and over 6.1% as under critical condition ^50^. These manifestations usually appear within 2 to 14 days after exposure and common signs include acute respiratory symptoms such as fever, cough, and difficulty breathing. In severe cases, COVID-19 symptoms include pneumonia, ARDS, kidney failure, and even death. The severity of symptoms is influenced by the immune system, age, certain comorbidities such as hypertension, diabetes mellitus, asthma, heart disease, obesity, and some habits such as smoking, lack of exercise, and staying in poorly ventilated rooms ^51^.

### Diagnostic test

The incubation period from when the virus was initially contracted to manifesting the first symptoms is usually 5 to 7 days (or within the range of 4–14 days). Current infection diagnosis relies on tests to detect the virus in various bodily fluids. Previous infections are confirmed through blood tests, and negative tests presume immunity to re-infection, although the duration and effectiveness of this protection are still unknown ^52^.

Laboratory-based molecular tests for detecting SARS-CoV-2 in the respiratory specimens are the current reference standard used to diagnose COVID-19, although serological immunoassays are rapidly being developed ^53^. One example of detection used respiratory specimens was conducted in Independent and Assisted Living Community for Older Adults —Seattle, Washington ^46^. The detection of SARS-CoV-2 using nasopharyngeal swabs was carried out twice, precisely day-one and seven, on the staff members. The positive cases in the first round were isolated immediately using personal protective equipment irrespective of whether they showed no symptoms. Furthermore, in the second round, positive cases were also discovered among the people who did not have symptoms initially. This analysis needs to be carried out because positive cases are bound to be found in the housing of a group of elderly or nursing homes. Therefore, this examination need not be carried out only once ^46^. IgM detection and IgA detection were possible from days 3 to 6 after the onset of the symptoms, while IgG starts to emerge from days 10 to 18 ^54^. Consequently, rapid antibody test is not recommended by the World Health Organization (WHO) as the primary basis for diagnosis. Therefore, serologically negative patients still need to be observed and re-examined to be confirmed ^55^.

Various screening methods are used to detect COVID-19, such as rapid antigen and antibody tests. Early diagnosis of COVID-19 requires gradual tests such as a screening test by conducting a rapid antigen test a week earlier and an antibody test that needs to be confirmed by RT-PCR and serological tests in the second week of COVID-19. Based on this study, the accuracy of most diagnostic tests such as RT-PCR, ELISA, POC LFA, CLIA, CEFA, and MIA the sensitivity and specificity increased in the late phase (>14 days) after the onset of symptoms. This accuracy helps identify individuals who have been exposed to COVID-19.

### Strengths and weaknesses of this study

This study reported that all COVID-19 tests are effective when carried out in accordance with their purpose and objectives. However, not all studies reviewed have a similar pattern. Therefore only a few were compared. Meanwhile, the cost of rapid testing was not discussed, which varies widely and allows for differences in the examinations' quality.

## Conclusion

The accuracy of rapid antigen tests remains debatable; therefore, RT-PCR should be preferred unless not available as the first-line strategy Finding new COVID-19 cases during this pandemic situation is extremely necessary to aid early detection with proper and mass surveillance. Therefore, treatments can be quickly administered and the source of transmission reduced. Tests for COVID-19 are generally divided into two, namely targeting the virus RNA and protein. The PCR method is targeted for RNA, while rapid tests for antigens and antibodies are targeted for proteins. The accuracy of these tests is supported by the sampling method from the incubation, emergence of symptoms, and healing period. Furthermore, the exposed individuals' contact history or positive case is also a significant factor determining sampling time with the appropriate type of test. The WHO recommends a rapid antigen test as an alternative supposing PCR is not available, therefore interfering with the handling of COVID-19 patients and the pandemic response process ^56^. Meanwhile, the rapid antigen test is effective when the number of cases is high because it detects virus material directly after symptoms. The result is known faster than the PCR test, compared to the rapid antibody test that increases in the second and third weeks after the onset of symptoms. Therefore, the order starts from the PCR test, then supposing it is unavailable, the rapid antigen test serves as an alternative when compared with the antibody test. However, the diagnosis should be confirmed using the PCR. Based on this study, the accuracy of most diagnostic tests such as RT-PCR, ELISA, POC LFA, CLIA, CEFA, and MIA sensitivity and specificity is increased in the late phase (> 14 days) after the onset of symptoms. This accuracy is helpful in the identification of individuals that have been exposed to COVID-19. To achieve an early diagnosis of COVID-19, according to disease progression, gradual rapid tests can be used, such as rapid antigen in an earlier week and antibody tests confirmed by RT–PCR and serological assay in the second week of COVID-19.

## Data availability

### Underlying data

All data underlying the results are available as part of the article and no additional source data are required.

### Reporting guidelines

Figshare: PRISMA checklist for 'A systematic review on the case findings and management of COVID-19; in the link https://doi.org/10.6084/m9.figshare.13586081.v1 ^8^

Data are available under the terms of the Creative Commons Attribution 4.0 International license (CC-BY 4.0).
