# Supplementary material for: A systematic review of the case findings, testing and management of COVID-19
Source: F1000Res. 2022 Aug 8;10:377. Originally published 2021 May 12. [Version 3] doi: 10.12688/f1000research.50929.3 (PMC9194520; doi:10.12688/f1000research.50929.3)
Supplement: Peer review history

# Peer review: Reviewer response for version 2

**DOI:** 10.5256/f1000research.79887.r125177

**Published:** 2022-06-13

**Authors:** Rahayu Lubis

**Affiliations:** 1 Faculty of Public Health, Universitas Sumatera Utara, Medan, Indonesia

I have read the paper entitled “ A systematic review of the case findings, testing, and management of COVID-19”. This paper is good and clear and beneficial for public health. The literature search for journal articles and Systematic Review stages is in accordance with standards.

 There are comments from me that do not affect the quality of this paper as written below:

1. At the end of the methods, it is written that the category of article quality assessment in percent is divided into bad (0-30%), satisfactory (34-67%), good (68-100%), there is a missing range, namely 31%, 32%, and 33% should be included in which category?
2. In the results section, it states that "The reactive results of patients examined by the rapid tests showed that 42.9% were asymptomatic or lacked available data". This needs attention because it is very dangerous for public health. COVID-19 patients are asymptomatic and can transmit COVID-19 to other people in their various activities.
3. "According to Table 3, showing the screening tests used in the study, 62% of the articles (13/21) adopted a rapid antibody and antigen test, for most of the men over 50 years of age" - what is the possible reason this test is less used at younger ages? This needs further research.

Are the rationale for, and objectives of, the Systematic Review clearly stated?

Yes

Is the statistical analysis and its interpretation appropriate?

Yes

Are sufficient details of the methods and analysis provided to allow replication by others?

Yes

Are the conclusions drawn adequately supported by the results presented in the review?

Yes

Reviewer Expertise:

Epidemiology and infectious disease

I confirm that I have read this submission and believe that I have an appropriate level of expertise to confirm that it is of an acceptable scientific standard.

# Response

**Published:** 2022-06-30

**Authors:** Dewi Susanna

**Affiliations:** Universitas Indonesia, Indonesia

1. At the end of the methods, it is written that the category of article quality assessment in percent is divided into bad (0-30%), satisfactory (34-67%), good (68-100%), and there is a missing range, namely 31%, 32%, and 33% should be included in which category?

*Response:*

*Thank you for the correction.*

*We are very sorry about the wrong classification. Now, it is corrected either in the methods and the note of Table 3: bad (0-33%)*

 2. In the results section, it states that "The reactive results of patients examined by the rapid

 tests showed that 42.9% were asymptomatic or lacked available data". This needs attention

 because it is very dangerous for public health. COVID-19 patients are asymptomatic and can

 transmit COVID-19 to other people in their various activities.

*Responses:*

*Thank you for your attention:*

*In order to avoid misunderstanding among readers, we* *break down the data in table 4 and added a statement* *“* *that 14.3% were asymptomatic and 19% not available data* *”.*

 3. "According to Table 3, showing the screening tests used in the study, 62% of the articles

 (13/21) adopted a rapid antibody and antigen test, for most of the men over 50 years of

 age" - what is the possible reason this test is less used at younger ages? This needs further

 research.

*Responses:*

*Thank you for your attention:*

*In order to avoid misunderstanding among readers, we deleted it. We added a statement that* *“62% of the articles used a rapid antibody test for screening rather than a rapid antigen test. According to the rapid antigen test, 51.3% were positive, with men aged above 50 years recording the highest number of cases* *”.*

**For the previous comment: Version 1**

 This article is useful and provides information about the COVID-19 early detection test but does not discuss the cost of the test.

*Responses:*

*Thank you for your concern.*

*Yes, you are right. It is a good idea to discuss the cost of the tests used since the costs vary. Unfortunately, in this study, we do not discuss the cost of COVID-19 testing; However, this is a good idea for suggestions in future studies considering the cost of the covid tests around the world, such as in the airports. For this issue, we added one reference, no 56.*

 Thank you very much for your valuable comments and corrections. 

 Best regards.

# Peer review: Reviewer response for version 2

**DOI:** 10.5256/f1000research.79887.r122044

**Published:** 2022-03-07

**Authors:** Sonu Menachem Maimonides Bhaskar

**Affiliations:** 1 Liverpool Hospital, Liverpool, NSW, Australia; 2 Ingham Institute, Liverpool, NSW, Australia; 3 Global Health Neurology Lab and NSW Brain Clot Bank, Sydney, NSW, Australia

The revised manuscript has significantly improved.

 No further comments.

Are the rationale for, and objectives of, the Systematic Review clearly stated?

Yes

Is the statistical analysis and its interpretation appropriate?

Partly

Are sufficient details of the methods and analysis provided to allow replication by others?

Partly

Are the conclusions drawn adequately supported by the results presented in the review?

No

Reviewer Expertise:

COVID-19, Public Health, Epidemiology

I confirm that I have read this submission and believe that I have an appropriate level of expertise to confirm that it is of an acceptable scientific standard.

# Peer review: Reviewer response for version 1

**DOI:** 10.5256/f1000research.54024.r94254

**Published:** 2021-10-04

**Authors:** Sonu Menachem Maimonides Bhaskar

**Affiliations:** 1 Liverpool Hospital, Liverpool, NSW, Australia; 2 Ingham Institute, Liverpool, NSW, Australia; 3 Global Health Neurology Lab and NSW Brain Clot Bank, Sydney, NSW, Australia

This is an interesting review of the case findings, testing, and management of COVID-19. The article needs substantial revision for its content. Here are some general comments for the authors to consider:

1. In the Abstract, the authors state "This review aimed to examine the outcome of the existing studies on the ways of identifying COVID-19 cases...", please revise this to "..examine the variations in COVID-19 diagnostic testing and clinical characteristics across various studies.". The study doesn't provide data on management protocols or on the outcomes.
2. The Discussion lacks a concrete summary. Please summarise the findings relevant to this systematic review.
3. Table 1 should be provided as a part of the Results.
4. In Table 2, the data on clinical characteristics, such as risk factors, COVID-19 severity, etc., are not provided and could be included. Moreover, please change the legend of Table 2 to "Clinical and demographic characteristics of studies included in the systematic review".
5. In the Discussion, "High-risk group" section, the authors make statements that are not appropriately referenced and lacks context from the standpoint of the overall findings of this review.
6. The authors discuss the symptoms in Table 4; however, several studies have not reported on various symptoms or data is simply not available?
7. The authors conclude "The elderly and individuals with pre-existing medical conditions such as high blood pressure, heart and lung disorders, diabetes, and cancer are at greater risk of experiencing severe COVID-19 symptoms.". But this is not supported by the findings of this study. The authors didn't examine these associations in this systematic review.
8. The Conclusion and Discussion need substantial revision focussing on the findings of this systematic review only. The accuracy of rapid antigen tests remains debatable, therefore, unless not available, RT-PCR should be preferred as the first-line strategy.
9. The authors need to expand upon the statistical analysis, as in what descriptive statistics were used for subgroup analyses.

Are the rationale for, and objectives of, the Systematic Review clearly stated?

Yes

Is the statistical analysis and its interpretation appropriate?

Partly

Are sufficient details of the methods and analysis provided to allow replication by others?

Partly

Are the conclusions drawn adequately supported by the results presented in the review?

No

Reviewer Expertise:

COVID-19, Public Health, Epidemiology

I confirm that I have read this submission and believe that I have an appropriate level of expertise to confirm that it is of an acceptable scientific standard, however I have significant reservations, as outlined above.

# Response

**Published:** 2021-11-28

**Authors:** Dewi Susanna

**Affiliations:** Universitas Indonesia, Indonesia

Dear editor and reviewer,

 Here are our responses to the reviewer comments and suggestions: 

 1. In the Abstract, the authors state, "This review aimed to examine the outcome of the existing studies on the ways of identifying COVID-19 cases...", please revise this to "..examine the variations in COVID-19 diagnostic testing and clinical characteristics across various studies.". The study doesn't provide data on management protocols or on the outcomes.

**Author Responses:**

 The aim of this abstract and the last part of the introduction was revised as follows:

*The review aimed to examine the variations in COVID-19 diagnostic testing and clinical characteristics across various studies*

 2. The Discussion lacks a concrete summary. Please summarise the findings relevant to this systematic review.

**Author Responses:**

 The revision of the Discussion is as follows:

 a. The section High-Risk Group was changed with Commorbidies and added for the new Discussion:

**Comorbidities:**

*COVID-19 patients with other comorbidities such as chronic obstructive pulmonary disease (COPD), cardiovascular disease (CVD), hypertension, cancer, diabetes, HIV, chronic kidney disease can cause a high risk of death. Comorbidities cause COVID-19 patients to be more at risk of increased morbidity and mortality 42-45. A cohort study in Jakarta also found a higher risk of death with comorbid patients than those without, the risk increasing sixfold among patients <50 years of age 46. Therefore, comorbidities can exacerbate COVID-19 infection 47*.

 Here are the added references, so the reference list must be reordered.

 41. Ejaz H, Alsrhani A, Zafar A, Javed H, Junaid K, Abdalla AE, et al. COVID-19 and comorbidities: Deleterious impact on infected patients. J Infect Public Health [Internet]. 2020 Dec 1 [cited 2021 Nov 27];13(12):1833–9. Available from: https://pubmed.ncbi.nlm.nih.gov/32788073/

 42. Sanyaolu A, Okorie C, Marinkovic A, Patidar R, Younis K, Desai P, et al. Comorbidity and its Impact on Patients with COVID-19. SN Compr Clin Med. 2020;2(8):1069–76.

 43 Fathi M, Vakili K, Sayehmiri F, Mohamadkhani A, Hajiesmaeili M, Rezaei-Tavirani M, et al. The prognostic value of comorbidity for the severity of COVID-19: A systematic review and meta-analysis study. PLoS One [Internet]. 2021;16(2 February):1–25. Available from: http://dx.doi.org/10.1371/journal.pone.0246190

 44. Surendra H, Elyazar IR, Djaafara BA, Ekawati LL, Saraswati K, Adrian V, et al. Clinical characteristics and mortality associated with COVID-19 in Jakarta, Indonesia: A hospital-based retrospective cohort study. Lancet Reg Heal - West Pacific [Internet]. 2021;9:100108. Available from: https://doi.org/10.1016/j.lanwpc.2021.100108

 45. Klein F. Risikofaktor Komorbiditäten bei COVID-19- Erkrankung. Pneumologie. 2020;74(10):640.

 b. The findings were summarised to relevant review:

 The last part of the Discussion summarised the review as follows:

*Various screening methods are used to detect COVID-19, such as rapid antigen and antibody tests. Early diagnosis of COVID-19 requires gradual tests such as a screening test by conducting a rapid antigen test a week earlier and an antibody test that needs to be confirmed by RT-PCR and serological tests in the second week of COVID-19. Based on this study, the accuracy of most diagnostic tests such as RT-PCR, ELISA, POC LFA, CLIA, CEFA, and MIA the sensitivity and specificity increased in the late phase (>14 days) after the onset of symptoms. This accuracy helps identify individuals who have been exposed to COVID-19.*

 3. Table 1 should be provided as a part of the Results.

**​​​​​​​Author Responses:**

 Table 1 moved to the results. It is put after Figure 1.

 4. In Table 2, the data on clinical characteristics, such as risk factors, COVID-19 severity, etc., are not provided and could be included. Moreover, please change the legend of Table 2 to "Clinical and demographic characteristics of studies included in the systematic review".

**​​​​​​​Author Responses:**

 The legend of Table 2 is replaced with: “ *Clinical and demographic characteristics of studies included in the systematic review”*

 5. In the Discussion, "High-risk group" section, the authors make statements that are not appropriately referenced and lacks context from the standpoint of the overall findings of this review:

**​​​​​​​Author Responses:**

 The section **High-Risk Group **was changed with **Comorbiditie**s and the Discussion added information as shown in responses No. 2.b above.

 6. The authors discuss the symptoms in Table 4; however, several studies have not reported on various symptoms or data is simply not available?

**​​​​​​​Author Responses:**

 The characteristics of the symptoms shown are following the results of the journals reviewed.

*7. *The authors conclude "The elderly and individuals with pre-existing medical conditions such as high blood pressure, heart and lung disorders, diabetes, and cancer are at greater risk of experiencing severe COVID-19 symptoms.". But this is not supported by the findings of this study. The authors didn't examine these associations in this systematic review.

**​​​​​​​Author Responses:**

 The findings *: "The elderly and individuals with pre-existing medical conditions such as high blood pressure, heart and lung disorders, diabetes, and cancer are at greater risk of experiencing severe COVID-19 symptoms” *was **deleted** since it is not supported by the data and the authors did not measure the associations.

 8. The Conclusion and Discussion need substantial revision focussing on the findings of this systematic review only. The accuracy of rapid antigen tests remains debatable; therefore, unless not available, RT-PCR should be preferred as the first-line strategy.

**​​​​​​​Author Responses:**

*‘The accuracy of rapid antigen tests remains debatable; therefore, RT-PCR should be preferred unless not available as the first-line strategy’ *was put **in the first line **of the conclusion.

 9. The authors need to expand upon the statistical analysis, as in what descriptive statistics were used for subgroup analyses:

**​​​​​​​Author Responses:**

**​​​​​​​**

 The authors did not use statistical analysis, for instance, a meta-analysis, because quantitative data such as Odds ratio, p-value, etc. were not supported in the articles selected.
